# Supplementary material for: Acceptance and compliance with micronutrient powder (MNP) among children aged 6–23 months in northern Nigeria
Source: PLOS Glob Public Health. 2022 Oct 17;2(10):e0000961. doi: 10.1371/journal.pgph.0000961 (PMC10022258; doi:10.1371/journal.pgph.0000961)
Supplement: S1 Dataset — (ZIP) [file pgph.0000961.s008.zip › S8_Dataset/In-depth Interviews.zip!/In-depth Interviews/070715_A_GA_BA_2_CG.pdf]

**INDEPTH INTERVIEW (IDI) WITH MRS REJOICE OF GANYE 1 WARD COMMUNITY, GANYE L.G.A ON 07/07/2015**

Introduction

My name is Rejoice and I'm a married woman living with my husband and only son which is 8 months old and currently on MNP feeding. Food for young children is never a challenge regardless of the season because we have varieties of food in this community. Also, children under the age of 2 years don't require much varieties because all they require is breast milk and light foods like kunu and tea mostly and little of solid meal as introduction into the food available in the community. Finance as well can't be a problem because my husband like most household in this community owns a farmland where we cultivate varieties of crops especially maize and guinea corn which are ingredients for kunu. Also, we store the crops so that we can have them in stock all through the season and they are equally cheap in the market. Seriously food for young children is not a challenge. It becomes a challenge later in life when varieties are required and the children are much to look after. When the children are much, they may not have the quantity of food needed for their satisfaction and the food may not be rich enough such as fish may not be in the meal, they may have 2 meals in a day instead of 3 meals and so on.

Caregivers often give their children tea because children love sweet things and prefer liquid foods. Apart from tea, children are in high demand for fruit and juice as well. For me, I don't give my son tea for now because the breast milk and cerelac food is adequate. Sometimes, I do give him herbal preparation

against diarrhea. It's in liquid form and I have to force him to take it because it's bitter in taste and he won't accept it willingly. The cerelac food is a processed food made of maize and it's in powdery form. I fetch the quantity he can finish and add hot water to it then stir to make a paste. The MNP is then added to the paste and stir to have a fine mixture before feeding the child with it. The cerelac is very easy to prepare and he likes it a lot. In most cases, he finishes the quantity I made for him but whenever it remains, I preserve it till when next his hungry. I dislike wastage and I was told the food containing the MNP must be completely consumed by the baby to ensure full effectiveness of the product.

On the positive aspect, he has added weight compared to before we started giving him the MNP. Also the MNP has increased his eating habit. Initially I use to give him one cup of cerelac meal but he now takes one and half or two cups of the meal. He's more active and hardly falls ill like his use to be.

My challenges using the MNP are:

i. Initially I use to pour all the MNP powder in one sachet at once into the cerelac. I observed that it doesn't mix well instead it formed balls on the cerelac and it requires constant mixing to break the balls so as to obtain a fine mixture. After a while, I discovered the best way to have a fine mixture without stress is to pour the MNP gradually while stirring till all the MNP in the sachet is completely added to the cerelac.

ii. At the initial stage when we started giving the MNP to the boy, his stool became black coupled with diarrhea and vomiting. I was disturbed and hard wanted to stop feeding him with the MNP but the father

encouraged me to continue. But after a while it subsided and it has stopped for now. So I advised that a small pamphlet indicating the action of the drugs and side effects should be inserted into the sachet so that one will not panic if such side effects occur because we were worried when the diarrhea started up till the extent that we had to take the child to the hospital to explain to the doctor which further advice us not to worry and assured us that the product is not harmful based on the contents of the product.

No problem with storing the MNP because it is stored in the drawer and it's very safe there. Whenever I want to use it, I simply open the drawer to bring it out. No kids around the house to tamper with it and rodents or any animal can't access it there.

Before the introduction of MNP, we hard several advice on good foods needed for the healthy growth of our child such as soya beans mixed with dried fish and so on but my elder sister and the hospital doctor I normally visit asked me to stick to breast milk mainly which is adequate for now and when I started using the MNP, they advice me to continue that its good for the child.

Though I used to give him the herbal preparation almost on daily basis to prevent the diarrhea, for the diarrhea related to the MNP, I had to give him flagil (it's anti-diarrhoea). I don't use to lack it in my house. Whenever I observe changes in his stool, I give him little of it. I don't give him always. It's been days I last gave him the flagil since his stool has returned back to normal. Though diarrhea is common with some foods started at the initial stage before the child's body get's use to it.

I have never forgotten to give him the MNP. Immediately I wake up from sleep, I reach out to his special cup and spoon to prepare his food before he starts crying. The MNP is always beside his food because I consider it as part of his food. I give the MNP at anytime of the day. It could be morning, afternoon or evening. I only make sure it's given to him once in a day.

My advice to people with forgetful attitude towards using the MNP for their baby is that, this MNP is for the benefit of your child and several challenges had been undergone to get this product down to use. So, it's important for the caregivers or mothers like me to give their baby so as to improve their health. Caregivers should try to make it as a point of first duty while waking up in the morning. Just as you remember your child every day and night, you should remember the MNP in that manner. I consider it as a food because it's multivitamin. So, caregivers should resist forgetfulness and should use the MNP for their child and use it appropriately as recommended.

Apart from cerelac, I give him soup just a little for him to taste while I'm eating just to introduce him gradually to food. From the instruction on the big sachet, a child I suppose to take all the content of the small sachet every day, so I ensure it goes that way.

The child doesn't share the MNP but I have to taste the food containing the MNP to know if it's too hot or warm enough before giving the child. Outside that, nobody else share from it.

On whether the 2 big sachets is enough, ill prefer the child to continue feeding on the MNP till he's 2 years of age. Since I know the worth of the MNP, I'll buy it if it's to be sold. I don't know the amount used to

manufacture the MNP, so I'll only make a random guess on how much it should be sold for the manufacturer not to enquire lost and for the people to be able to afford it. If I should consider the angle of the people especially myself, I believe 1,000 naira will be fine for the 2 sachet.

My senior sister influences my decision on child feeding. Whenever I have issue regarding that, I call her for advice. I lived with that my sister during my childhood. Also I have my family doctor that I seek her advice regarding child feeding. she's based in yola and I call her whenever there is problem. On advice relating to the use of MNP, its people like me (the interviewer) and any other personnel working on the MNP programme. That is why it's important for her to collect my number before I leave so that she can contact me whenever there is problem to seek my advice.

On place to give health education regarding MNP, radio, antenatal care unit in hospitals and house to house will be the best channels to reach out to masses. On house to house, not everyone will be accommodating. Some can even collect and just dump it aside without using it. The radio such as the fm radio is a good source of reminder and it can come as a jingle which is much better.

On other programmes like yours that succeeded e.g immunization against diseases such as poliomyelitis, they suffered before gaining acceptance. They all passed through this processes you are undergoing now. Like in poliomyelitis, some people rejected initially due to some misconception that it's intended to reduce population. You should expect rejection by some people. Don't be discouraged because with constant persuasions, people now seek for immunization up to the extent that some people have

to pay for it and form long queue at the hospital. Though yours will not go through that long phase of suffering/rejection because the level of awareness has improved drastically and people are now embracing innovations and quality live even at the rural level.

On distribution, the card system should be used just like in mosquito net distribution. While the caregiver keeps the card, the distributor will hold the register so that the card will be ticked whenever there is distribution till the end of the whole phase of distribution. The distribution will be done every 2 months, since the 2 big sachets will be given. The house to house distribution using the card system is the best. Since much population is require to be reached, the antenatal care unit at various health facilities should also be used just the way nursing mothers go for immunization at the hospital and get their attendance paper ticked. Transparency is require to avoid some people from getting surplus while others not getting at all.

On message to carry for acceptance, you should first greet the household members on getting to there and then introduce yourself and also the organization you represent as well as the importance of the MNP for the children. Try to sound responsible while you give all necessary explanations and if possible go there with a familiar face the people know like the way you did while coming to me. Had it been you came alone, I wouldn't have accepted you easily because I don't know you and everyone is security conscious these days. If rejection happens by using house to house, compliment with using the antenatal care unit of the hospital. Though some people don't go to the hospital and even give birth at home. So using the traditional birth attendant can equally reach such people.  The local radio channels such as the fm yola should be used e.g. immunization against

poliomyelitis passed through this process and people are now aware that they are seriously sourcing for the poliomyelitis vaccines.

On advice against sharing of the MNP, it's the mother that feeds the child since a child of this age range can't feed himself. The mother should feed the child separately before he goes to join his siblings to continue feeding during meal time.

My questions are:

i. Should mothers feed on the MNP and pass it through breast milk to the child?

Answer: the MNP is meant for the child and the method of passing it through breast milk is never an effective means for the MNP.

ii. Let's say I travelled and spent somedays without giving the child MNP, is it advisable to continue the MNP when I returned back?

Answer: we discourage forgetfulness because skipping days of administration will reduce the effectiveness of the MNP for the child. On whether to continue, yes you can continue provided the child is within the age range of 6 to 23 months.

iii. Let's say I have 3 neighbours and I'm the only person that received the MNP for my child. After explaining to them on the importance of the MNP to the child while they saw me feeding my child with the product, is it advisable to share with them if they request? Because if I resist to share with them, they will feel offended. It will look as if I

don’t care for their own children as well and I’m being selfish. That may bring enemity between us which I dislike.

Answer: the MNP is not meant to be shared. What was given to you is meant for one child within the age range of 6 to 23 months only. Though we’ll soon carry out a wide distribution so that those your friends will benefit and issue of that sharing will not occur. So try to keep those your friends aware when the distribution starts so that they won’t depend on the portion meant for your child alone.
